# Association between patient-reported cognitive function and location of glioblastoma

**DOI:** 10.1007/s10143-023-02177-z

**Published:** 2023-10-25

**Authors:** Stine Schei, Lisa Millgård Sagberg, Lars Eirik Bø, Ingerid Reinertsen, Ole Solheim

**Affiliations:** 1 Department of Public Health and Nursing, Norwegian University of Science and Technology, Mauritz Hansens Gate 2, 7030 Trondheim, Norway; 2 Department of Neurology, St. Olavs hospital, Trondheim, Norway; 3 Department of Neurosurgery, St. Olavs hospital, Trondheim, Norway; 4 Department of Health Research, SINTEF Digital, Trondheim, Norway; 5 Department of Circulation and Medical Imaging, Norwegian University of Science and Technology, Trondheim, Norway; 6 Department of Neuromedicine and Movement Science, Norwegian University of Science and Technology, Trondheim, Norway

**Keywords:** Patient-reported outcome measures, Cognition, Surgery, Magnetic resonance imaging, Glioblastoma, Voxel-based lesion-symptom mapping

## Abstract

Objective cognitive function in patients with glioblastoma may depend on tumor location. Less is known about the potential impact of tumor location on cognitive function from the patients’ perspective. This study aimed to investigate the association between patient-reported cognitive function and the location of glioblastoma using voxel-based lesion-symptom mapping. Patient-reported cognitive function was assessed with the European Organisation for Research and Treatment (EORTC) QLQ-C30 cognitive function subscale preoperatively and 1 month postoperatively. Semi-automatic tumor segmentations from preoperative MRI images with the corresponding EORTC QLQ-C30 cognitive function score were registered to a standardized brain template. Student’s pooled-variance *t*-test was used to compare mean patient-reported cognitive function scores between those with and without tumors in each voxel. Both preoperative brain maps (*n* = 162) and postoperative maps of changes (*n* = 99) were developed. Glioblastomas around the superior part of the left lateral ventricle, the left lateral part of the thalamus, the left caudate nucleus, and a portion of the left internal capsule were significantly associated with reduced preoperative patient-reported cognitive function. However, no voxels were significantly associated with postoperative change in patient-reported cognitive function assessed 1 month postoperatively. There seems to be an anatomical relation between tumor location and patient-reported cognitive function before surgery, with the left hemisphere being the dominant from the patients’ perspective.

**Supplementary Information:**

The online version contains supplementary material available at 10.1007/s10143-023-02177-z.

## Introduction

Tumor location is an important factor to consider in surgical decision-making in glioblastoma patients, and a potential determinant of both preoperative objective cognitive impairment and perioperative changes [1–4]. The prevalence of cognitive deficits in glioblastoma patients ranges from 22 to 100% in different studies [5]. Structures involving language function, the basal ganglia, corpus callosum, cingulate cortex, and hippocampus are traditionally considered important for cognitive functions [6]. A range of advanced pre- and intraoperative tools may assist surgeons in identifying and minimizing the risk of damage to perceived eloquent regions [7–9]. However, which brain regions are the most important for cognitive function from the patients’ perspective is less known. A few previous studies have explored the relationship between patient-reported cognitive function and glioma location, but as a secondary outcome measure focusing on distinct and larger regions, such as lobes and lateralization [10–13]. Consequently, potentially important regions for patient-reported cognitive function may be overlooked [14]. In addition, since there is no consensus on how to define tumor location, it is often based on arbitrary criteria, which hampers objectivity and comparison of results. Voxel-based brain maps of three–dimensional (3D) segmented tumors can overcome some of these limitations and give a more accurate measure of tumor location.

In this population-based cohort study, we aimed to investigate the potential impact of tumor location on preoperative and postoperative change in patient-reported cognitive function in patients with glioblastoma using voxel–based lesion–symptom mapping (VLSM).

## Materials and methods

### Study population

Adult glioblastoma patients (≥ 18 years) scheduled for primary resection or biopsy only between September 2011 through December 2020 were eligible for inclusion. The patients were retrospectively identified from a regional brain tumor database at the Department of Neurosurgery, St. Olavs hospital, Trondheim, Norway. This department serves a defined geographical catchment region with approximately 750 000 inhabitants. The patients underwent surgery under general anesthesia, and the tumor was histopathologically classified as glioblastomas according to the 2007 or 2016 World Health Organization classification [15, 16]. Exclusion criteria were missing preoperative patient-reported cognitive function, known dementia, and/or missing preoperative magnetic resonance imaging (MRI) scans. Patients operated in several sequences (i.e., multifocal resections), undergoing biopsy only, and/or missing postoperative cognitive function score were included in the study of preoperative maps, but excluded from the maps of postoperative change.

### Variables and data collection

Patient-reported cognitive function was reported by the patients themselves or with assistance from a nurse or family member 1–3 days before surgery and approximately one month after surgery (median 34 days, range 19–63 days) with the European Organisation for Research and Treatment (EORTC) QLQ-C30 cognitive function subscale (Norwegian translated) [17]. The EORTC questionnaire is a 30-items questionnaire that comprises a global quality of life scale, five functional scales, and six single items. The cognitive function subscale is one of the functional scales and includes the following two questions: During the past week: “have you had difficulty remembering things?” and “have you had difficulty in concentrating on things, like reading a newspaper or watching television?.” The questions are answered on a four-point scale from “not at all” to “very much.” The answers to these two questions were converted into a cognitive function score ranging from 0–100, with higher scores indicating better cognitive function [18].

The Karnofsky Performance Status (KPS) was scored prospectively by the operating surgeon [19]. In cases of missing KPS score (*n* = 3), medical notes were used to retrospectively estimate if the patients were functionally dependent (< 70) or independent (≥ 70). Patient- and treatment characteristics were retrieved from electronic medical records. Comorbidity was scored according to Charlson Comorbidity Index (CCI) [20], and postoperative complications within 30 days were graded according to the Landriel classifications system [21].

### Brain imaging and segmentation

MRIs were routinely acquired < 72 h before surgery with a 1.5 or 3 Tesla MRI scanner. Tumor volumes were estimated by semi-automatic 3D tumor segmentation using the software packages 3D Slicer version 4.3.1–4.11 (3D Slicer, Boston, Massachusetts) [22] and BrainVoyager QX version 1.2 (Brain Innovation, Maastricht, Netherlands). We have previously demonstrated high agreement between these software packages [23]. In contrast-enhanced lesions, tumor volume was defined as pathological contrast enhancement and necrotic tissue within the contrast-enhancing borders seen on T1-weighted images (*n* = 156). In non-enhanced lesions, fluid attenuation inversion recovery images were used (*n* = 6). Images were segmented as part of several previous studies in glioblastoma. Several junior doctors/PhD students were trained in image interpretation by a neuroradiologist or an experienced glioma surgeon (OS) and image segmentations were reviewed by an neuroradiologist or the same neurosurgeon (OS). The extent of resection was calculated from pre- and postoperative MRI images as the relative postoperative reduction of the preoperative tumor volume in percentage and further dichotomized as gross total resection (100%) or subtotal resection (< 100%). In one patient, postoperative computed tomography (CT) images were used to determine subtotal extent of resection. Lateralization was categorized according to where the center of mass in each tumor was located, while multifocal bilateral tumors were categorized as a separate group.

### Brain maps and statistical analyses

As described in a previous publication [24], the preoperative MRI segmentations were spatially aligned with the standardized frame of reference known as the Montreal Neurological Institute (MNI) space, defined by the ICBM-152 brain template [25]. Two sets of tumor maps were then created: one based on the preoperative EORTC cognitive function scores, and one based on the changes in function scores from preoperative to postoperative scoring. Each set consisted of three different maps: A distribution map showing the number of patients with a tumor in a given voxel, a statistical map showing voxels with a statistically significant correlation between the presence of a tumor and function score based on VLSM, and a descriptive map showing the mean cognitive function score for patients with tumor in a given voxel.

The VLSM analysis was performed using the NiiStat toolbox for Matlab (http://www.nitrc.org/projects/niistat). Here, for each voxel with at least three tumors, the patients were divided in two groups: those with tumor in the given voxel and those without. A Student's pooled-variance t-test was then performed to compare the cognitive scores between the two groups. The significance threshold was set to *p* ≤ 0.05. Then, since the test was performed for a large number of voxels, a permutation method [26] using 2000 permutations was applied to correct for multiple comparisons. Finally, the corrected threshold was applied to the map, retaining only voxels with statistically significant Z score.

SPSS Statistics version 28.0 (IBM, Armonk, New York) was used for descriptive statistics. Patient-, treatment- and disease characteristics are presented as either median with range or frequencies.

## Results

**Fig. 1:**
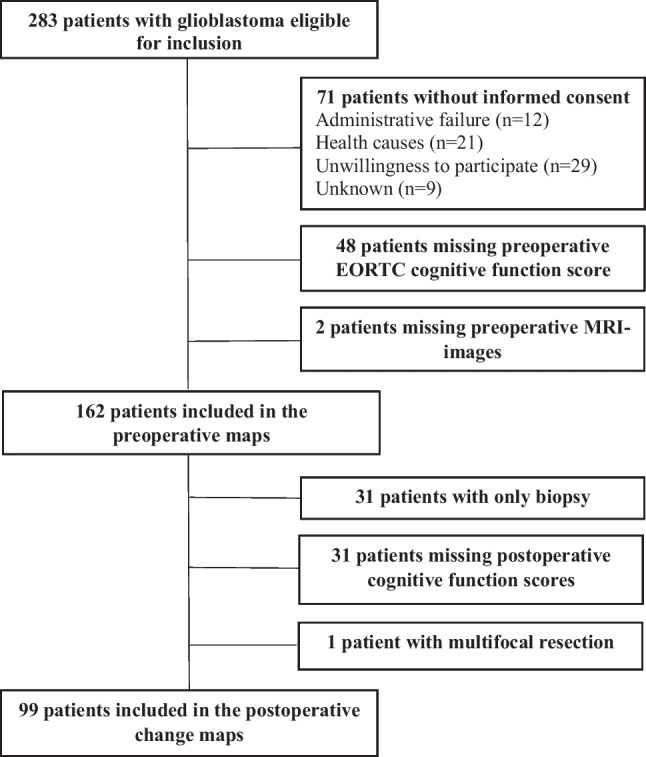
Flowchart of the inclusion process

In total, 162 patients with glioblastoma were included in the preoperative brain maps (both resections and biopsies), and 99 patients were included in the postoperative change maps (resections only). The inclusion process is presented in Fig. 1.

### Baseline and treatment characteristics

Baseline and postoperative treatment and disease characteristics are presented in Table 1. As seen, the median age was 62 years (range 29–83 years), and most patients were functionally independent with KPS ≥ 70 before surgery (86%). There were 74 glioblastomas (46%) located in the left hemisphere, 85 (52%) were right sided, while 3 (2%) were bilateral. There was no significant difference in preoperative tumor volume between the hemispheres (*p* = 0.14). Gross total resection was achieved in 31% of the patients, and 87% had initiated oncological treatment within one month of follow-up.

**Table 1:** Baseline-, treatment-, and disease characteristics

Baseline characteristics (n = 162)	
Age in years, *median* (range)	62 (29–83)
Female sex, *n* (%)	55 (34)
Preoperative Karnofsky Performance Status score ≥ 70, *n* (%)	139 (86)
Preoperative symptoms, *n* (%)	
Headache	65 (40)
Seizures	49 (30)
Nausea/vomiting	25 (15)
Unsteadiness/ataxia	57 (35)
Language	48 (30)
Visual	15 (9)
Cognitive	66 (41)
Motor	44 (27)
Charlson Comorbidity Index ≥ 2, *n* (%)	7 (4)
Tumor lateralization, *n* (%)	
Left	74 (46)
Right	85 (52)
Bilateral	3 (2)
Preoperative tumor volume ml, *median* (range)	26.6 (0.96–159.7)
Preoperative use of corticosteroids, *n* (%)	139 (86)
Preoperative use of antiepileptic drugs, *n* (%)	48 (30)
Treatment and disease characteristics after resection (n = 99)	
Gross total resection (100%), *n* (%)	32 (32)
Postoperative new or worsened motor/language deficits before discharge,* n* (%)	14 (14)
Landriel grade II-III complications within 30 days, *n* (%)	10 (10)
Postoperative radio- and/or chemotherapy within one month follow-up, *n* (%)	86 (87)

### Brain maps with preoperative patient-reported cognitive function

**Fig. 2:**
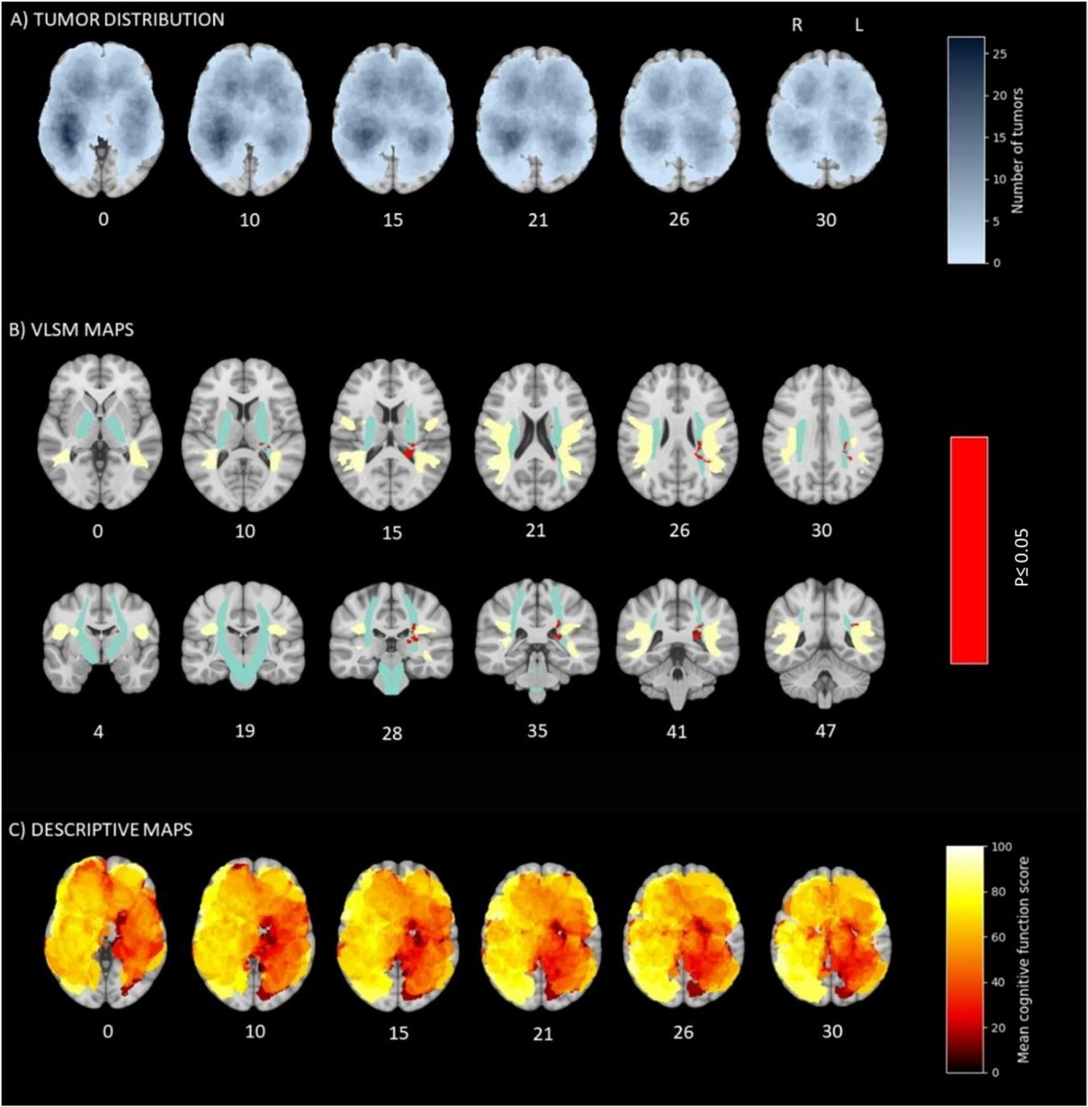
Preoperative patient-reported cognitive function. The number under each cross section shows its coordinate in the ICBM-152 brain template [25]. **A** Number of tumors in each voxel, with darker blue indicating more tumors. **B** Voxels with a statistically significant correlation between the presence of tumor and cognitive function score (red) with atlas of arcuate fasciculus (yellow) and corticospinal tract (turquoise) for reference. The significance threshold of *p* ≤ 0.05 corresponded to a Z score < -4.91 after permutation correction. **C** Mean preoperative cognitive function in each voxel, with darker red indicating more cognitive problems

Maps of preoperative tumor distribution, preoperative VLSM maps, and descriptive maps with mean preoperative patient-reported cognitive function, are presented in Fig. 2 and in Video 1 (Online Resource 1). The red spots in the VLSM maps show that several regions in the left deep central hemisphere were statistically significantly associated with preoperative patient-reported cognitive symptoms. The significant voxels were seen in the superior part of the left lateral ventricle, the lateral part of the left thalamus, the left caudate nucleus, and in a portion of the internal capsule just medial to the left arcuate fasciculus. Also based on the descriptive maps is seen that patients with tumors in the left hemisphere report worse preoperative cognitive function than patients with corresponding tumors in the right hemisphere. Particularly, tumor location in the left central structures, including the posterior part of the corpus callosum, the cingulate gyrus, hippocampus, and basal ganglia, seems to be linked to worse function, although not all regions were significant in the VLSM analyses.

### Brain maps with postoperative changes in patient-reported cognitive function

**Fig. 3:**
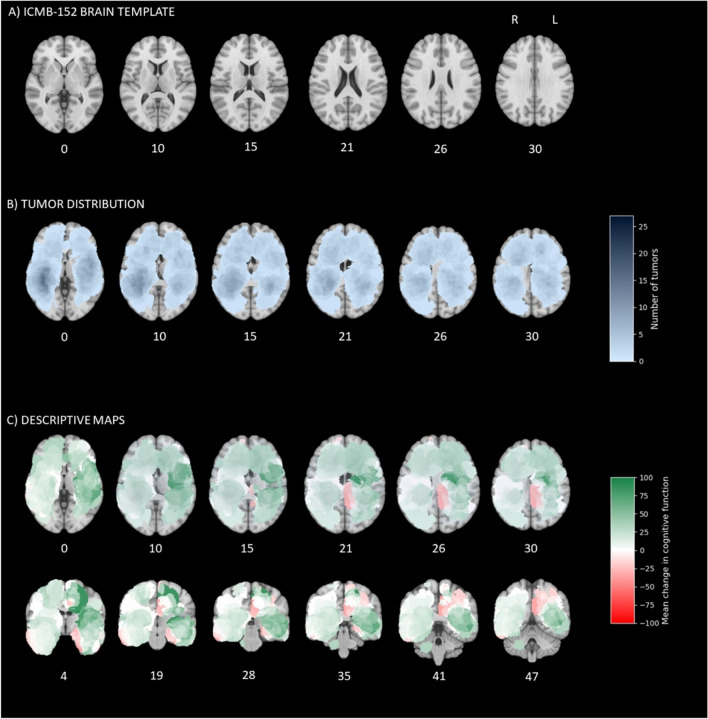
Postoperative change in patient-reported cognitive function. The number under each cross section shows its coordinate in the ICBM-152 brain template [25]. **A** The ICBM-152 brain template for reference. **B** Number of tumors in each voxel, with darker blue indicating more tumors. **C** Mean postoperative change in cognitive function in each voxel, with green indicating improvement and red worsening

For patients who underwent surgical resection and with postoperative EORTC cognitive function score (*n* = 99), brain maps of preoperative tumor distribution and descriptive maps with mean change in patient-reported cognitive function at one month are presented in Fig. 3 and in Video 2 (Online Resource 2). The VLSM analysis of postoperative cognitive change was not significant, and hence no VLSM maps were created. From the descriptive maps, postoperative worsening of cognitive symptoms is seen in the left central hemisphere, including the posterior cingulate gyrus, and an area near the hippocampus. In contrast, patients harboring right hemisphere tumors more often reported unchanged or improved cognitive function.

## Discussion

This study used the VLSM method to examine the potential importance of tumor location for patient-reported cognitive function in a fairly large population-based sample of newly diagnosed glioblastoma patients. We found voxels in the left hemisphere to be significantly associated with worse preoperative cognitive function, including the lateral part of the left thalamus, the left caudate nucleus, and the left internal capsule, medial to the left arcuate fasciculus. Significant voxels were also found inside the left lateral ventricle, probably caused by inaccuracies in the registration of periventricular tumors due to mass effect and/or edema. Patient-reported cognitive function may be a relevant measure, and there seems to be an anatomical relationship between patient-reported cognitive function and tumor location.

Traditionally, the left hemisphere has been viewed as the most important and respected due to its dominance in language processing [27], and patients with left-hemispheric glioma are found to have more objective cognitive impairment than patients with right-sided glioma [2–4]. Our findings suggest that also from the patients’ perspective, the left hemisphere of the brain may be more dominant for cognitive function. We found an accumulation of significant voxels near the arcuate fasciculus, which connects the frontal, parietal, and temporal lobes and plays an important role in language processing [28]. Although language function was not formally tested in our study, language is presumed to play a central role in human cognition. It may therefore be difficult, also for the patients themselves, to separate language difficulties from other cognitive problems. Furthermore, we found an accumulation of significant voxels in the left internal capsule and the left lateral thalamus. Since the internal capsule contains fiber tracts coordinating cognitive pathways and the lateral thalamus relays limbic functions [29], more problems in these areas seem logical. Rather similar results are also found in other glioma studies [30, 31] and previous studies of objective cognitive function in stroke patients [32, 33]. Significant voxels were also seen in the caudate nucleus, which has a role in memory, learning, and executive functioning [34]. Another explanation for the left hemisphere's dominance may be that the right hemisphere plays a special role in cognitive functions that may not necessarily be picked up in the EORTC questionnaire, such as processing nonverbal information and perceptions of the body in relationship to the trunk and surrounding space [6].

Hemispheric distinctions in affective and emotional responses to brain damage may also explain the differences in patient-reported cognitive function between left-sided and right-sided tumors. Catastrophic reactions and stronger emotional responses to illness, as well as depression and anxiety, appear to be more frequent in patients with left hemispheric injury [35, 36]. Although many glioma studies have found no association between depression and hemispheric laterality [37], methodological limitations prevent definite conclusions [37, 38]. On the contrary, damage in the right hemisphere has been linked to a lack of awareness of mental abilities (anosognosia) [39], which may lead the patients to underestimate their true abilities. However, a study of high-grade glioma patients found that many patients are aware of their cognitive deficits after treatment with no difference in tumor laterality [12].

Postoperative improvement in glioblastoma patients may be a result of reduced mass effect, reduced peritumoral edema, termination of high-dose corticosteroids, psychological factors and more. However, the VLSM analysis of postoperative patient-reported cognitive change yielded no significant voxels, indicating that cognitive change may not be as location specific as the preoperative cognitive state. Still, from the descriptive maps, there seem to be several locations linked to postoperative improvement, except for the left central structures, where postoperative worsening was more often observed. A higher likelihood of surgery-induced damages in these areas may explain our finding. An alternative explanation might be that neurosurgeons have a more conservative surgical approach to centrally located tumors due to their potential critical impact on outcomes. However, our descriptive findings must be interpreted cautiously, and additional studies are needed to better understand these findings.

Cognitive function has been widely studied in neuropsychological tests [2, 4, 40], but the test results often differ from the patients’ experience [11, 41]. In this study, cognitive function was measured with the patient-reported questionnaire EORTC cognitive function subscale to ensure relevance for the patients. The outcome measure does not replace a neuropsychological test, but the subscale represents an approach to obtain outcome data about the patient’s self-perceived cognitive function. Patient-reported cognitive function was operationalized by the two questions about concentration and memory. Thus, we were not able to measure locations vulnerable to specific mental abilities, and a more comprehensive questionnaire may have detected more subtle impairments and yielded more detailed results. Also, despite the fairly large sample size, there were too few tumors in some voxels to be included in the analyses, and thus not all structures important for patient-reported cognitive function might have been detected. Still, there seems to be an anatomical relation between tumor location and self-reported cognitive function in glioblastoma patients, supporting a potential clinical validity of patient-reported measures of cognition.

The fairly large prospectively collected population-based sample is a major strength in this study, increasing the generalizability of our findings. Still, selection bias may have occurred, given some patients' lack of informed consent or nonresponding at 1 month follow-up. This is an unavoidable issue in glioma studies [42]. Furthermore, other factors important for patient-reported cognitive function may have affected our results, such as the use of corticosteroids and antiepileptics, oncological treatment, and tumor progression. Also, we did not register handedness, but the left hemisphere is dominant in 95% of right-handers and 70–80% of left-handers [6]. Another limitation is that several people contributed with tumor segmentations, which might have influenced the results. However, everyone who contributed has been trained in image interpretation by a neuroradiologist or an experienced glioma surgeon and image segmentations were reviewed by a neuroradiologist or the same neurosurgeon.

Although the VLSM method is increasingly used to measure the role of tumor location to functions, it has some disadvantages. First, registration of MRI images to the standardized MNI space can cause inaccuracies, especially in cases of significant mass effect and/or edema. Second, the tumors may functionally affect regions outside their radiological borders due to mass effects, edema, and their infiltrating and rapid tumor growth. Third, the heterogeneous distribution of tumors within the brain means that statistical power in many voxel-based analyses may be low. Since VLSM results can be vulnerable to false negatives in regions with few tumors, we decided to include descriptive maps. However, descriptive maps should be interpreted with caution. Still, as also argued by others, VLSM is the best available method to assess the relationship between location and functioning in brain tumor patients and allows valid conclusions [43].

## Conclusion

We found the left hemisphere to be dominant for preoperative cognitive function from the patients’ perspective. More specifically, glioblastomas around the superior part of the left ventricle, the left lateral thalamus, the left caudate nucleus, and the left internal capsule by the arcuate fascicle were significantly associated with reduced patient-reported cognitive function before surgery. No areas were found to be significantly associated with patient-reported postoperative changes. Our findings suggest that there might be an anatomical relationship between patient-reported cognitive function and tumor location.

### Supplementary Information

Below is the link to the electronic supplementary material.

Supplementary Video 1 Axial maps of preoperative tumor distribution, descriptive maps with mean preoperative patient-reported cognitive function, and preoperative VLSM maps. N = 162 (MP4 23917 KB)

Supplementary Video 2 Axial maps of preoperative tumor distribution for the sample with postoperative follow-up and descriptive maps with mean postoperative change in patient-reported cognitive function at 1 month. N = 99 (MP4 12868 KB)

## Data Availability

The dataset generated during and/or analyzed during the current study are not publicly available due to privacy concerns but are available from the corresponding author on reasonable request.
